# Introducing insect- or plant-based dinner meals to families in Denmark: study protocol for a randomized intervention trial

**DOI:** 10.1186/s13063-022-07000-6

**Published:** 2022-12-20

**Authors:** Cassandra Maya, Luís Miguel Cunha, Ana Isabel de Almeida Costa, Teun Veldkamp, Nanna Roos

**Affiliations:** 1 Department of Nutrition, Exercise and Sports (NEXS), University of Copenhagen, Rolighedsvej 26, 1958 Frederiksberg, Denmark; 2 GreenUPorto/Inov4Agro, DGAOT, Faculdade de Ciências da Universidade do Porto, Porto, Portugal; 3 Universidade Católica Portuguesa, CATÓLICA-LISBON School of Business & Economics, Palma de Cima, 1649-023 Lisboa, Portugal; 4 Wageningen Livestock Research, P.O. Box 338, 6700 AH Wageningen, the Netherlands

**Keywords:** Acceptability, Alternative proteins, Dietary intervention, Edible insects, Meat replacement, Plant-based foods, Sustainable diet

## Abstract

**Background:**

Plant- and insect-based foods are promising alternative protein sources. Previous studies have shown that introducing plant-based foods to the diet can reduce meat intake, but no such intervention has explored the effects of insect-based foods.

**Methods:**

This study aims to integrate alternative proteins to main meals of 80 Danish families through a 6-week two-arm randomized intervention trial to investigate acceptance, consumption, and potential for meat replacement. The primary outcome is the replacement of dietary meat protein with plant- or insect-based protein from the intervention foods assessed through change in daily meat protein intake, proportion of meat protein to total protein intake, and counts of dinner meals with meat and intervention products.

**Conclusion:**

The results of this study will contribute to research in alternative proteins and explore the effects of long-term exposure of meat replacement.

**Trial registration:**

ClinicalTrials.gov: NCT05156853. Registered 24 December 2021

## Background

Dietary shifts to more sustainable food choices in the daily diet can make an important contribution to reach the target of reducing the environmental impact and greenhouse gas emission in Denmark. Overall, emissions for Nordic diets are estimated yearly at 1.9–2.0 tonnes CO_2_ equivalents with animal products being responsible for about 65–75% of these emissions [1]. Dietary shifts to reduce meat consumption relies on the consumer’s willingness to replace the consumption of meat with that of more sustainable protein sources.

Plant-based diets are viewed by society as a suitable alternative to reduce food-related environmental impact and have gained popularity in the past couple of years. Global retail sales of plant-based meat reached $4.2 billion USD in 2020, up from $3.4 in 2019 [2]. The EU Green Deal’s Farm to Fork strategy promotes the creation of a resilient, safe, and affordable sustainable food system that responds to consumer demands for healthy and environmentally friendly products [3]. Similarly, the Intergovernmental Panel on Climate Change describes sustainable healthy diets as those that “promote… health and well-being; have low environmental pressure and impact; are accessible, affordable, safe and equitable; and are culturally acceptable” and specifically refers to plant-based foods as a part of this diet [4]. The Danish Veterinary and Food Administration recommends eating a plant-rich diet of fruits, vegetables, and legumes while reducing the intake of meat for healthier and more sustainable food consumption [5]. A modified version of the EAT-Lancet diet to better fit Danish dietary guidelines was nutritionally adequate for 6–65 year old individuals, with the exception of vitamin D and iodine [6].

Insects have become another promising alternative protein for human consumption and animal feed with a market estimate of $9.6 billion USD by 2030 [7] and recent legislative evaluations creating a clearer market path for insect-based products. The European Food Safety Authority (EFSA) has evaluated dried yellow mealworms (*Tenebrio molitor*), house crickets (*Acheta domesticus*), and buffalo worms (*Alphitobius diaperinus*) to be safe for human consumption [8–12]. Despite physiological differences between species, edible insects provide the nutritional benefits of animal-based foods [13, 14] with high protein quality [15] and less environmental impact (e.g., less water and land use) than traditional livestock [16], particularly when compared to beef and potentially less than chicken [17]. Additionally, insects can be reared with organic waste materials, such as food waste, distiller’s dried grains, and beer spent grains [18, 19].

Barriers to the consumption of edible insects are complex; low familiarity, food neophobia, and cultural unacceptability drives views of skepticism and disgust [13, 20–25]. Several factors can also influence acceptability and willingness to eat insects, such as age and previous exposure [26–29]. Insect-based food products are mainly marketed as exotic snack products, like whole dried flavored insects, and protein bars which may not be sufficient to encourage integrating insects as a meat replacement. One study showed that very few consumers moved from occasionally tasting insects to regularly eating them [30]. An influx of studies further explore acceptance strategies in varying populations, such as priming [31] and exposure [27, 32–34], as well as improving the sensory profile [35].

Food neophobia is not exclusive to insect-based foods. People often associate meat-inclusive dishes with tradition, values, and a part of complete meal [36]. Possidónio et al. [37] found committed meat eaters showed aversion towards plant-based substitutes, but meat eaters who were concerned with health and nutrition viewed plant-based substitutes (with the exception of lab grown meat) as healthier than red meat.

Interventions with plant-based alternatives have resulted in reduction of meat intake and changes to psychosocial variables, such as increased positive attitudes and reduced meat attachment [38, 39]. Introducing novel proteins for meat replacement presents numerous barriers that can influence how the food is received, such as lack of reliable information, knowledge and cooking skills, and social support [40]. To address this such obstacles, a plant-based menu will act as a control in the study to investigate if the effects of an insect-based dietary intervention differ from other alternative protein sources.

Sustainable Insect Chain (SUSINCHAIN) Work Package 5 aims to develop insect-based foods suited for integration in regular main meals in Europe and to investigate their acceptance, consumption, and potential to replace meat in diets, relative to that of extant plant-based protein sources.

### Objective and hypothesis

The overall objective of this study is to investigate the impact of exposing families (paired participants—an adult and a child) to dinner menus of meals with alternative proteins (insect-based or plant-based products) on dietary pattern, intake of meat, and protein over a 6-week intervention period. The insect-based menu is the experimental exposure, and the plant-based menu is the positive control menu.

The hypothesis is that test menus of meals with alternative proteins will replace the meat consumed during dinner, resulting in maintaining the total protein intake while replacing 20% of the meat protein with alternative protein on a weekly basis. The assumption is that the insect-based menus will replace meat protein similarly or to a larger extent than the positive control group receiving the comparable plant-based menu. The inclusion of the positive control group allows us to isolate the specific impact of exposure to insect-based menu from the exposure to dietary change of more familiar plant-based products. The protein replacement is determined by tailoring the protein content of the experimental products included in the menus of three weekly portions of the alternative protein products to national average levels of meat intake. The assumption is that meat is primarily consumed for lunch and dinner meals.

#### Primary outcome

The primary outcome is the replacement of dietary meat protein with protein from the intervention foods (insect- or plant-based). The co-primary endpoints are changes in the intake of meat protein over the 6-week intervention period and the measures for replacement by the intervention products. The change is assessed as (1) change in intake of total daily amount of meat protein at baseline (prior to receiving the intervention products) and endline (sixth weeks of receiving the intervention products), (2) change in proportion of meat protein of the total protein intake, and (3) counts of dinner meals with meat and alternative protein products during baseline week 0 and during each of the intervention weeks, including the endline assessment during the 6th week of intervention.

#### Secondary outcome

The secondary outcome is change in the sensory evaluation of the intervention foods. The secondary endpoint is changes in the sensory parameters for liking of the intervention food prepared as meals.

The hypothesis is that the liking and acceptance of the novel products will increase over time [27, 41, 42]. Demographic and anthropometric measures, as well as other individual-level characteristics (degree of food neophobia and food disgust, earlier exposure to edible insects), are included as explanatory variables.

## Materials and methods

### Study design

The study is a two-arm randomized equivalence intervention trial. The participants are recruited from families. In each family, one adult and one 8–10 year-old child are enrolled as paired participants. The families are provided products incorporating alternative proteins (insect-based or plant-based) suited to replace meat in dinner meals three times per week, for 6 weeks.

**Fig. 1:**
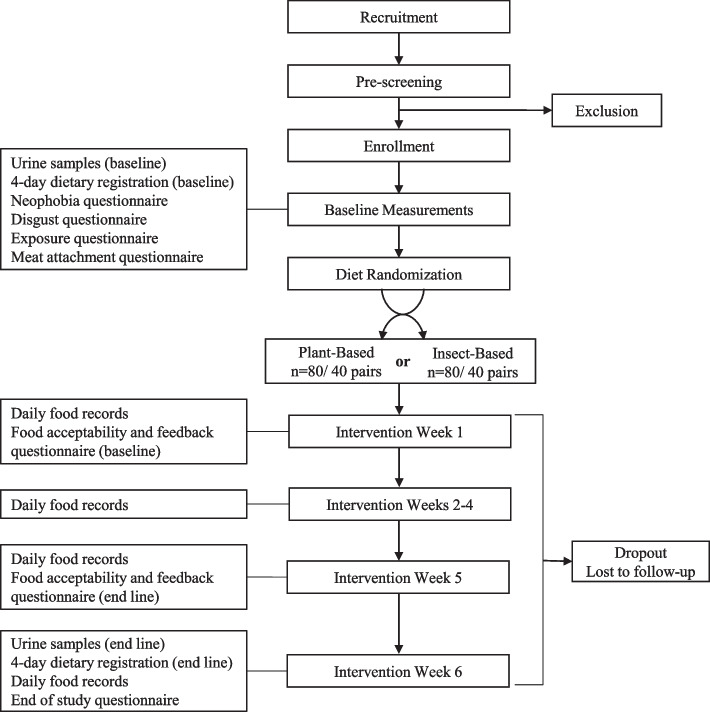
Flowchart of the study design

Figure 1 depicts the study flowchart. Recruitment, information meetings, collection of informed consent, and other relevant duties are conducted by the on-site project management team and supervised by the PI. During the recruitment period, interested parents and children will attend an information meeting at the Department of Nutrition, Exercise and Sports of the University of Copenhagen, formally enrolling to participate when signing consent. Subsequently, participants receive instructions for baseline measurements. Baseline measurements are completed during the following week, to estimate habitual protein intake (urine and dietary registrations), food neophobia (questionnaire), food disgust (questionnaire), and previous exposure to edible insects (questionnaire). Participating adults will complete a household characteristics questionnaire and a meat attachment questionnaire. One week later, the participants are returning to the department for random allocation to experimental diets, and distribution of products for the following 2 weeks. During that visit, anthropometric measurements will be collected prior to the start of the dietary intervention. Daily food records (questionnaire) will start during the first week of the dietary intervention up to the final day of the dietary intervention. Intervention week 1 includes the baseline food acceptability and feedback questionnaire, to be completed by the participating adult and child. Intervention week 5 includes the endline food acceptability and feedback questionnaire. During intervention week 6, the paired participants will collect endline urine samples and complete the endline 4-day dietary registrations and end-of-study questionnaires. To aid with retention, reminders are sent to participants to complete the registrations, questionnaire, and prior to appointments.

### Intervention menu

Three study food products will be delivered per week over the 6-week study period. One of two menus (insect-based or plant-based) will be assigned to the paired participants. A randomly assigned sub-menu (a, b, or c) will determine the order of foods throughout the study. During the first 2 weeks of the study, participants will try each of the six selected food products from their assigned menu. In the following weeks, the assigned sub-menus will provide the same number of meals for each of the food products, with only the assigned order differing between the sub-groups.

#### Portion sizes

The menus are composed of packed portions estimated to on average replace 20% meat protein intake for one adult and one child calculated on a weekly basis using data from 2011–2013 Danskernes Kostvaner [43]. The menus of either insect-based products or plant-based products are provided to the paired participants (one adult and one child) as packed portions dimensioned to provide the amounts of protein set to reach the targeted protein replacement. If family members not enrolled as participants want to be part of the dinner meal, additional portions are provided on request. Based on the assumption that the meat consumption is equally distributed between lunch and dinner meals, the national average amounts of meat protein per meal is set to 13.4 g for adults and 9.7 g for children. The insect-based or plant-based products are provided in portion sizes providing insect protein matching the average meat consumption to provide a full replacement of meat regularly consumed for the three weekly dinner meals.

#### Insect-based foods

Six insect-based products were designed and developed by partners in the SUSINCHAIN project. Production occurred in the appropriate food manufacturing facilities, approved for this type of production. The products include chili-tomato paste (incorporating *Acheta domesticus*) from Bugging Denmark (Copenhagen, Denmark), crispbread (incorporating *A. domesticus*) from Marche Polytechnic University (Ancona, Italy), falafel (incorporating *Alphitobius diaperinus*) from New Generation Nutrition (Den Bosch, Netherlands), minced meat (incorporating *Tenebrio molitor*) from KU Leuven (Leuven, Belgium), spice mix (incorporating *A. domesticus*) from the Technical University of Denmark (Lyngby, Denmark), and sausages (incorporating *A. domesticus* and *A. diaperinus*) from LEITAT (Barcelona, Spain). Criteria was set by the partners so that the developed products were visually and texturally appealing by avoiding whole insects and being familiar and convenient to consumers. Products were subject to sensory testing by project partner Sense Test (Vila Nova de Gaia, Portugal) before being approved for the intervention.

#### Plant-based foods

The plant-based menu will consist of market available products. The plant-based products matched as closely as possible the insect-based products in terms of presentation and nutritional composition. To avoid influence from branding, packaging will be altered.

### Study participants

#### Inclusion criteria

Participants must be healthy, which is determined from the self-reported medical history. All medication that is necessary for the participant’s health or a medical condition and which is not listed in the exclusion criteria may be continued during the study.

Participants must eat meat for dinner at least 5 days a week on average and be willing to consume both insect- and plant-based foods. Participants must be able to talk, read, and understand Danish to properly understand the study procedures.

Adults must have basic computer competency and the household must have freezer capacity for 2 weeks of food.

Children must be between 8 and 10 years old. If a child wants to participate, but their custody holder does not want to participate, another adult from the household can participate with the child. The custody holder is still responsible for the child’s participation in the study.

#### Exclusion criteria

Participation in other clinical studies is not allowed throughout the entire intervention study. Participants must not possess any food or dust mite allergies or intolerances [44, 45]. Participants who follow one or more restricted diets (veganism, gluten-free, keto, vegetarianism, etc.) or use protein supplements do not qualify for this study. Participants with gastrointestinal, kidney or liver disorders, chronic inflammation disorders (excluding obesity), and systematic antibiotic use less than 1 month prior to the study will be excluded. Diagnosed psychiatric disorders, including depression, requiring treatment can be accepted based on judgment. Participants must not be pregnant or lactating within the study period or have a self-reported use of drugs of abuse (including cannabis) within 12 months prior to the study. Participants that lack the abilities (physical and psychological) to comply with the procedures in the protocol may be excluded.

#### Recruitment

Participants will be recruited from the Copenhagen metropolitan area of Denmark. Recruitment is conducted through announcements on social media, word of mouth, flyers, and project webpage. Additionally, children of the relevant age can be requested through the Danish Civil Registration System (CPR registry) and custody holders will be invited to the study. The adult of the household will be invited to participate, to be participating together with the child, as paired participants. Recruitment rate is between 5 and 15 families a month, depending on holiday and travel seasons.

#### Randomization and blinding

Randomization will be performed in blocks of six paired participants, to assure equal allocation to the order of the two menus over the recruitment period. A computer-generated randomization list with 80 consecutive numbers will be created and stored in opaque, sealed envelopes until assigned. The allocation and enrollment is conducted by authorized site personnel. Each participant will be assigned a participant identification number (ID) and diet at the first follow-up visit. Though each participant has a unique ID, the diet will be the same for the paired participants. Participants are randomly assigned to the intervention diet by a draw from the randomized, sealed envelopes. Due to the taste and nature of the intervention foods, blinding of certain study staff and participants is not possible after the first follow-up visit. Data assessors are blinded to diet allocation.

## Measurements

### Characteristics of food perception and exposure

#### Food neophobia

All participating adults will use the Food Neophobia Scale adapted from Pliner and Hobden [46]. The questionnaire has five added questions specifically for the insect-based foods menu and will use a 7-point anchored scale (1—totally disagree and 7—totally agree). The Food Neophobia Test Tool, adopted from Pliner [47] and Damsbo-Svendsen et al. [48], will be used for all participating children using a 7-point smile scale.

#### Disgust questionnaire

All participants will complete a modified version of the food disgust questionnaire designed by Hartmann and Siegrist [49], along with additional questions that specifically apply to edible insects as adapted from Rozin et al. [50]. The original items “The texture of some kind of fish in my mouth” and “There is a little snail in the salad that I wanted to eat” from the 8-item short version of the Food Disgust scale were replaced with “To eat fish like sushi” and “There is a worm in my apple” from the 32-item Food Disgust Scale, respectively, in order to adapt to Danish culture. Adults will use a 7-point anchored scale, with 1—not disgusting at all and 7—extremely disgusting, for the main questionnaire and the anchors 1—totally disagree and 7—totally agree for the additional questions. Children will use a 7-point smile scale.

#### Exposure to edible insects

A modified exposure questionnaire adapted from Verbeke [28] is specific to insect-based foods but will be administered to all participants prior to randomization. Participating adults and children will select the statement that best applies to them. The original item “I eat insects seasonally” was changed to “I’ve tried edible insects many times” to better suit the Danish population.

#### Meat attachment

Meat attachment will be evaluated using a modified version of the questionnaire created by Graça et al. [51]. This questionnaire will only be administered to participating adults using a 7-point anchored scale (1—totally disagree and 7—totally agree).

#### End-of-study questionnaire

The end-of-study questionnaire will record participant attitude towards alternative proteins and the study products post intervention, as well as general feedback towards the organization of the study. The questionnaire will be primarily completed by the adult but will have a few short questions at the end for the adult and child to answer together as well.

### Dietary measurements

#### Four-day dietary registration

A complete 4-day dietary registration at baseline and endline will be done for all participants. The dietary registration is conducted by the use of MyFood24, a dietary web-based software available in Danish (myfood24.org).

#### Daily food records

Once the study food is distributed, the participants will be asked to keep daily records of the consumption of the intervention food using a simple online questionnaire. Participants will note if they have consumed an intervention food and to specify which and the amount consumed. They will be asked if they ate meat that dinner and, if so, to specify the type and the amount consumed.

#### Food acceptability and feedback

Participants will be asked to record their liking of two intervention foods at the first exposure (baseline) and last exposure (endline). The two products will be assigned randomly per menu.

### Other measurements

#### Household characteristics and demographics

The participating adult will fill in a questionnaire about household characteristics, including household size, ethnicity, and education. General information (age, gender, education, and ethnicity) about other household members will be collected for characterization.

#### Anthropometrics

Standard anthropometric measures (height and weight) of all participants are assessed at the first follow-up visit to be able to validate energy and protein intake recorded in the dietary registrations. Height and weight will be measured using standard procedures from the department.

#### Urine collection

Protein intake will be assessed through urine analysis using methods adapted from Yuan et al. [52]. Urea nitrogen, or carbamide, will be measured using a potentiometer (ABX PENTRA 400, Horiba Medical, Japan) and creatinine levels will be used to normalize for dilution. Spot urine samples will be taken on every other day (for a total of 3 samples) of the week prior to consuming the study foods (baseline) and during the final week of the intervention (endline). The collected urine samples will be stored in a designated research biobank and only identifiable by the assigned participant ID. We will request consent to review participants’ urine samples to assess protein intake in the current trial through the GDPR consent form. Possible excess material can be transferred to a biobank for approved, new, and related research projects if the adult and custody holders (on behalf of the child) have consented separately. Otherwise, excess materials will be destroyed.

### Data management

Documents that identify the participant will be maintained in strict confidence by the principal investigator, except to the extent necessary to allow auditing and/or monitoring by the appropriate regulatory authority and research associates. All information obtained during the study will be handled according to local regulations and GDPR by qualified personnel.

### Statistics

In order to measure the 20% replacement of meat in meals with insect- or plant- based alternatives from *x̄* = 14 meals/week to *x̄* = 11.2 meals/week, we will need a sample size of 78 paired participants. This sample size is based on *α* = 0.05, *β* = 0.80, and an assumed *σ* = 7. The standard deviation of the protein intake in the target population is uncertain and the statistical power is therefore based on assumptions. The results of the study will have important scientific value independent of the assumption of statistical power.

Statistical analysis will be used to determine changes in meal patterns as counts of meals with meat and the total protein intake at baseline and endline both within and between the insect and plant groups, hypothesizing no change in total protein intake and 20% replacement of the meat meal using linear models. The same analysis will be conducted both within and between the meat proteins with the insect-based foods at baseline and endline. A Spearman’s rho test will be used to assess perception change (neophobia, exposure, and disgust) before and after the intervention. A paired sample *t*-test (or Wilcoxon test, if not normally distributed) will be used to analyze pre- and post- intervention urine samples.

### Risks, side effects, and drawbacks

Participating in a study can cause some inconvenience to daily life and can be time-consuming. There are no substantial risks associated with participation in the intervention. There is no anticipated harm and compensation for trial participation.

#### Safety of study foods

The study food products are processed, packaged, and stored by the EU partners in the SUSINCHAIN project. All products are produced in Europe. The products are processed, packed and stored in locations with facilities approved by relevant national food authorities. Samples of the insect-based foods are sent to a certified laboratory for microbial tests following general food standards.

#### Safety of study procedures

Spot urine collection is non-invasive and poses no risks to the participant.

#### Unexpected risks and monitoring

Rare cases of severe allergic reactions have been documented from edible insects, including anaphylactic shock. Allergic reactions to edible insects are often linked to existing allergies to crustaceans and dust mites. Persons with such allergies will not be included in the study. However, it cannot be excluded that an unforeseen reaction may occur [44, 45, 53]. Participants will be instructed to note any adverse events experienced during the study to report to the study staff. Participants will, at every visit, carefully be asked about adverse events by trained staff and will be documented on the correct form, including information on onset, severity, and end. The project sponsor is the University of Copenhagen and can be contacted through principal investigator NR (nro@nexs.ku.dk). The participants are insured in accordance with the law: “Bekendtgørelse af lov om arbejdsskadesikring” (LBK nr 376 af 31/03/2020) by the current insurance at University of Copenhagen (UCPH). During the study, the participants will be covered by the law: “Bekendtgørelse af lov om klage- og erstatningsadgang inden for sundhedsvæsenet” (LBK nr 995 af 14/06/2018).

Due to the low risk of the study, a formal data monitoring committee was not formed. There are no formal stopping rules for the trial because there are no anticipated problems that are detrimental to all participants.

The trial steering committee is composed of authors CM, LMC, AC, and NR and meets bi-weekly. Local organization, such as recruitment and enrollment, is conducted by the on-site study management team and supervised by the study PI. The on-site study management team will meet weekly to monitor the quality and progress of the intervention.

### Ethical aspects

#### Informed consent

Informed consent will include information on the study activities and procedures, right to withdrawal, collection and storage procedures, and data processing. The participating adult will sign on their own behalf and will be responsible for their own participation. The custody holders must be legally able to give written consent for the child’s participation and will be responsible for their child’s participation in the study and consent forms. Children will not be allowed to participate in the study without legal consent from their custody holders. In case of shared custody, both custody holders need to sign the informed consent, unless a power of attorney has been signed. The informed consent form is available from the corresponding author upon reasonable request.

#### Withdrawal

The participating adult as well as the custody holders can withdraw their consent for participation at any time without giving explanations and without any consequences for them or the child. If one member in the household withdraws, the other member may still participate. Both consent forms must be canceled individually.

The participants may be withdrawn at any point of the study if advised by the clinically responsible physician. The participating child may be withdrawn if it becomes evident that the child does not wish to be part of the study. Participants may be withdrawn from the study for non-compliance.

## Discussion

The results of this study contribute to the growing interest in promoting the inclusion of alternative proteins in the everyday diet. Improved insect acceptability through one-time or short exposures, such as information sessions, are documented [26, 29], but the effects of long-term exposure on reducing meat consumption remains unclear. To our knowledge, this is the longest insect protein intervention study to date that not only explores psychosocial measurements but delves into the potential of meat replacement.

This study presents several strengths. First, the study includes adult and child participants, which will highlight the differentiating perspectives that exist between age groups. Second, the products are catered to dinner meal options and surpass the scope of snack or novelty items. Third, the study is designed to directly compare with plant-based dietary intervention. Additionally, a similarly designed parallel study in Portugal is being undertaken for comparing across European populations. Limitations of the study include a limited number of participants.

## Trial status

The study protocol (version 2.0, 01 June 2022) was reformatted for submission for publication in scientific journal after receiving ethical clearance in June 2022. In case a change to the protocol is needed, an amendment application will be sent to the Danish National Committee on Health Research Ethics for review and approval. Recruitment began in August 2022 and is ongoing with an expected completion date of July 2023. Due to time allocation for implementing the study, our submission of the manuscript was the earliest possible. The results will be disseminated to interested persons through open-access publications, the SUSINCHAIN, and project website and their associated social media accounts.

## Data Availability

Any data required to support the protocol can be supplied on request. The full protocol for the current study are available from the corresponding author on reasonable request.

## Abbreviations

SUSINCHAIN: Sustainable Insect Chain
EFSA: European Food Safety Authority
GDPR: General Data Protection Regulation
